# Computationally Derived Points of Fragility of a Human Cascade Are Consistent with Current Therapeutic Strategies

**DOI:** 10.1371/journal.pcbi.0030142

**Published:** 2007-07-20

**Authors:** Deyan Luan, Michael Zai, Jeffrey D Varner

**Affiliations:** Department of Chemical and Biomolecular Engineering, Cornell University, Ithaca, New York, United States of America; The University of Tokyo, Japan

## Abstract

The role that mechanistic mathematical modeling and systems biology will play in molecular medicine and clinical development remains uncertain. In this study, mathematical modeling and sensitivity analysis were used to explore the working hypothesis that mechanistic models of human cascades, despite model uncertainty, can be computationally screened for points of fragility, and that these sensitive mechanisms could serve as therapeutic targets. We tested our working hypothesis by screening a model of the well-studied coagulation cascade, developed and validated from literature. The predicted sensitive mechanisms were then compared with the treatment literature. The model, composed of 92 proteins and 148 protein–protein interactions, was validated using 21 published datasets generated from two different quiescent in vitro coagulation models. Simulated platelet activation and thrombin generation profiles in the presence and absence of natural anticoagulants were consistent with measured values, with a mean correlation of 0.87 across all trials. Overall state sensitivity coefficients, which measure the robustness or fragility of a given mechanism, were calculated using a Monte Carlo strategy. In the absence of anticoagulants, fluid and surface phase factor X/activated factor X (fX/FXa) activity and thrombin-mediated platelet activation were found to be fragile, while fIX/FIXa and fVIII/FVIIIa activation and activity were robust. Both anti-fX/FXa and direct thrombin inhibitors are important classes of anticoagulants; for example, anti-fX/FXa inhibitors have FDA approval for the prevention of venous thromboembolism following surgical intervention and as an initial treatment for deep venous thrombosis and pulmonary embolism. Both in vitro and in vivo experimental evidence is reviewed supporting the prediction that fIX/FIXa activity is robust. When taken together, these results support our working hypothesis that computationally derived points of fragility of human relevant cascades could be used as a rational basis for target selection despite model uncertainty.

## Author Summary

To date, mechanistic mathematical modeling, in general, has not played a significant role in the development of new therapies for cancer, cardiovascular diseases, or the treatment of acute events like thrombosis during surgery. One critical issue often cited for the lack of interest has been uncertainty; the conventional wisdom is that the data requirement to fully determine and validate large mechanistic models is just too high. We show, using tools from systems biology and sensitivity analysis, that it may be possible to extract qualitative information about the critical elements of human relevant cascades despite model uncertainty. Using a mechanistic model of the human coagulation cascade, we were able to identify the critical mechanisms controlling the formation of thrombin, a key protein active in the formation of blood clots. We were further able to support the hypothesis that the critical mechanisms identified by our analysis could serve as drug targets by comparing our findings with the thrombosis treatment literature and with current clinical trials. The results support the notion that mechanistic models could be used, despite model uncertainty, to pinpoint key mechanisms in complex networks, and that these mechanisms could potentially be therapeutically exploited.

## Introduction

The role that mechanistic mathematical modeling and systems biology will play in molecular medicine and clinical development remains uncertain. Kitano suggested that understanding of critical questions in biology required the integration of experimental and computational research [1]. Assmus et al. and others maintained that analysis of the dynamics of human relevant networks using predictive computer models and high-throughput data generation would play an increasingly important role in medical research and the elucidation of disease mechanisms [2,3]. However, parametric and structural uncertainty remains an open challenge to mechanistic modeling in medicine.

Strategies that integrate experimental and computational techniques have had success at elucidating network structures. Arm and Arkin reviewed experimental and computational techniques to uncover molecular interaction networks [4]. The central experimental advancements in the area of protein–protein network identification have been the yeast two-hybrid system [5,6] and quantitative mass spectrometry proteomic techniques to determine protein complexes [7,8]. Young and coworkers explored protein–DNA interactions using the chromatin immunoprecipitation technique [9] where likely transcription factor binding sites were determined using a combination of chromatin immunoprecipitation chips and DNA microarrays. Time-lagged correlation matrices [10,11], genetic programming techniques [12], and network decomposition strategies have also been used with time-series concentration measurements to estimate reaction network structures [13].

Sensitivity analysis has been used to integrate model identification and discrimination with optimal experimental design and knowledge discovery. Cho et al. used sensitivity analysis to study TNF-α–mediated NF–κB signalling where parametric uncertainty was addressed using Monte Carlo sensitivity analysis; using the best-guess parameter set, a family of random parameter sets was generated where sensitivity coefficients were calculated for each member of the random family [14]. Cho et al. went on to develop a unifying framework, building upon the earlier work of Kholodenko et al. and Sontag et al. to unravel the functional interactions in biomolecular networks using a stimulus–response strategy and metabolic control analysis [15–17]. Kremling et al. investigated the benchmark problem of growth of a microorganism in a continuous bioreactor subject to feed shifts using sensitivity-based model identification and discrimination strategies; they determined optimal experimental design and perturbation strategies to identify and discriminate between rival model formulations [18]. Gadkar et al. identified signal transduction models from time-course measurements using a nonlinear scheme to estimate missing protein measurements from measured values [19]. They went further and proposed strategies to calculate D-optimal experimental designs that maximized the experimental information used to identify signal transduction models as well as an iterative strategy to explore model structure [19,20]. Sensitivity analysis has also been used to explore the robustness and fragility of metabolic and signaling networks. Robustness, the ability to maintain system performance in the face of perturbation and uncertainty, is a desirable feature in both biological as well as man-made networks, machines, and systems [21]. Conversely, fragility, i.e., extreme sensitivity to small perturbations, is a very undesirable trait that could lead to catastrophic system failure following seemingly innocuous perturbations, e.g., a Boeing 777 crashing because of minor software failures or microscopic alterations in a few integrated chips [22]. Stelling et al. reviewed several examples of robustness in biological networks [21], while Leibler first computationally predicted and later experimentally verified robust features of chemotaxis control networks [23,24]. Bullinger and coworkers explored the robustness of models of programmed cell death or apoptosis [25], while Stelling et al. computationally identified points of robustness and fragility, using Monte Carlo sensitivity analysis and overall state sensitivity coefficients, in models of circadian rhythm [26].

In this study, we use tools from systems biology, namely mathematical modeling and sensitivity analysis, to explore the working hypothesis that mechanistic models of human relevant cascades, despite model uncertainty, can be computationally screened for points of fragility, i.e., sensitive mechanisms, and that these mechanisms could serve as a rational basis for therapeutic target selection. We test our working hypothesis by computationally screening a mechanistic model of the well-studied coagulation cascade developed and validated from literature sources. After model validation, using 21 published datasets generated from two different quiescent in vitro coagulation models, we use Monte Carlo sensitivity analysis to computationally screen the model for sensitive mechanisms in the presence and absence of natural anticoagulants. We then contrast the predicted fragile mechanisms with literature to determine if they are consistent with experimental investigation, thereby proving or disproving our working hypothesis. While the current development is restricted to coagulation, the broader strategy is general and could be applied to an arbitrary network.

### A Review of the Coagulation Cascade

Coagulation, mediated by a family of serine proteases (factors) and a key group of blood cells (platelets), both of which are normally inactive in the circulation, is directly relevant to human health and has been suggested by Somogyi and Greller to be an ideal candidate for in silico drug discovery [27]. Insufficient coagulation is manifested in disorders such as haemophilia A (1 in 5,000 live births), haemophilia B (1 in 30,000 live births), or von Willebrand disease (1 in 1,000 live births) [28,29]. Conversely, unwanted clotting can be a serious complication following surgical intervention and is directly involved in coronary artery diseases, which collectively account for 38% of all deaths in North America [30].

**Figure 1:**
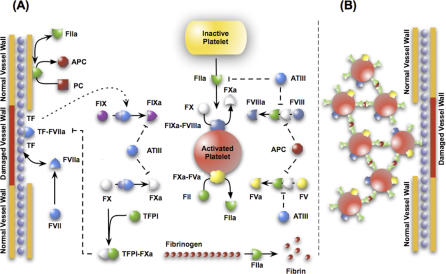
Schematic of the Extrinsic Coagulation Cascade (A) Upstream coagulation factors are activated by substances exposed by vessel injury; chief among these factors is TF. Activated upstream coagulation factors initiate a cascade of events that culminate in the activation of platelets and the key protease FIIa. Thrombin forms an amplification loop by activating itself and other coagulations factors as well as platelets. (B) Activated platelets then aggregate to form platelet plugs, which serve as scaffolds for fibrin clots.

The salient features of the coagulation cascade included in our model, shown schematically in Figure 1 and presented in detail in Table 1, are reviewed here. Several extensive reviews of the underlying biochemistry and cell biology of coagulation can be found elsewhere [31–34]. There are two pathways that lead to activation of the master protease thrombin and eventually to a clot—the intrinsic and extrinsic cascades. It is generally believed that the extrinsic cascade is the main mechanism of thrombinogenesis in the blood [33–35]. Upstream coagulation factors are activated by materials exposed because of vessel injury chief among these tissue factors (TFs) [36]; TF and activated factor VIIa (FVIIa) present in the blood form a complex that activates factor X (fX) and fIX. FXa activates downstream factors, including fV, fVIII, and fIX. FXa can also, along with FVa, form a complex on the surface of activated platelets that converts prothrombin (fII) to thrombin (FIIa). TF–FVIIa is not the only mechanism to activate fX; FIXa and FVIIIa can complex on the surface of activated platelets and catalyze the formation of FXa. Platelet localization at the wound site occurs through specific interactions between the platelet and the subendothelium, primarily through recognition of exposed materials such as collagen, fibronectin, and von Willebrand factor. Localized platelets are activated by external signals such as adenosine diphosphate and thrombin. Thrombin irreversibly activates platelets through a family of transmembrane receptors on the platelet surface called protease-activated receptors [37,38]. Thrombin, in addition to playing a key role in platelet activation, catalyzes the conversion of fibrinogen (secreted by activated platelets from internal stores) to fibrin. Fibrin, with the help of FVIIIa, forms a cross-linked mesh inside the platelet plug that stops blood flow. Thrombin also activates upstream coagulation factors, thereby forming a strong positive feedback that ensures rapid activation. Three control points that inhibit thrombin formation are considered in the model. TF pathway inhibitor (TFPI) downregulates FXa formation and activity by sequestering free FXa and TF–FVIIa in an FXa-dependent manner. Antithrombin III (ATIII) neutralizes all serine proteases generated during the coagulation response, making it perhaps the most powerful control element in the cascade. Thrombin itself plays an inadvertent role in its own inhibition by binding the surface protein thrombomodulin (TM), expressed on normal vasculature [39]. The FIIa–TM complex catalyzes the conversion of protein C (PC) to activated PC (APC); APC attenuates the coagulation response by the proteolytic cleavage of fV/FVa and fVIII/FVIIIa [39].

**Table 1:**
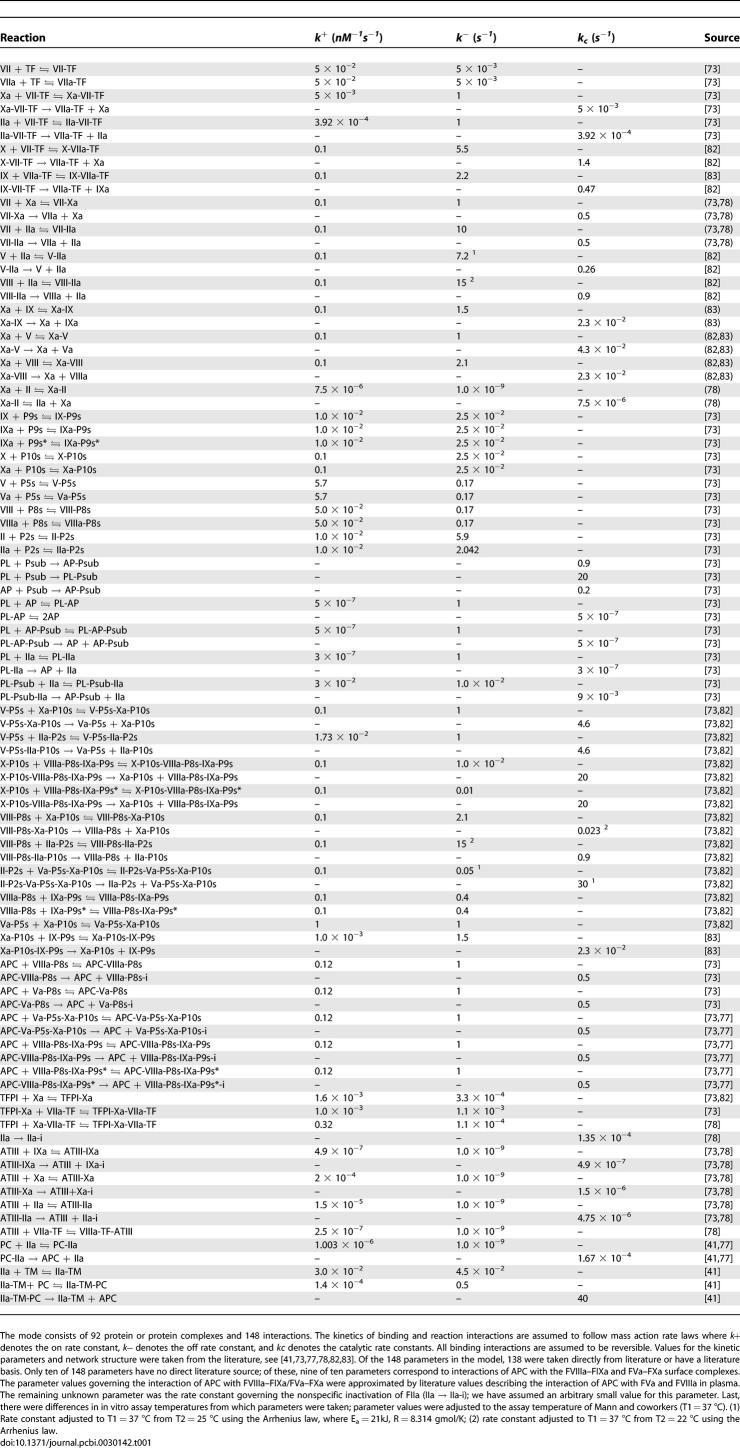
Reactions and Parameter Values Used in the Extrinsic Coagulation Model

## Results

**Figure 2:**
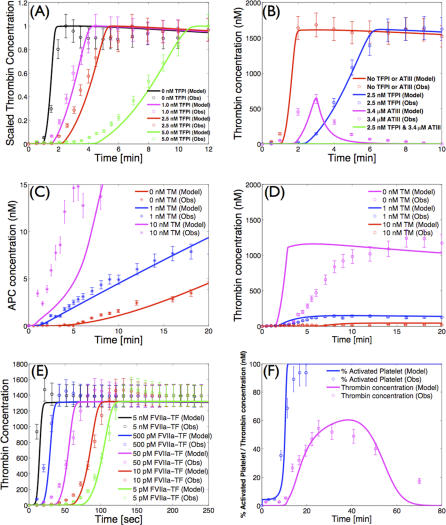
Model Validation Using Published In Vitro Datasets (A) Thrombin concentration versus time as a function of TFPI concentration following the addition of 1.25pMTF–FVIIa to synthetic plasma. (B) Thrombin concentration versus time for different combinations of TFPI and ATIII following the addition of 1.25pM TF–FVIIa to synthetic plasma. (C) APC concentration versus time as a function of TM concentration following the addition of 1.25pM TF–FVIIa to synthetic plasma. (D) Thrombin concentration as a function of time as a function of TM concentration following the addition of 1.25pM TF–FVIIa to synthetic plasma. (E) Thrombin concentration versus time as a function of TF–FVIIa initiation strength in synthetic plasma. (F) Fraction of activated platelets and thrombin concentration as a function of time in the cell-based assay. The synthetic plasma assay cases were reproduced from Mann and coworkers [31,41], while the platelet activation data in (F) were reproduced from Roberts et al. [40]. The GraphClick software (Arizona Software, http://www.arizona-software.ch) was used for data extraction where a coefficient of variation (CV) of ±10% was added to the data to account for extraction and experimental error. Initial conditions for all simulations are given in Protocol S1.

**Figure 3:**
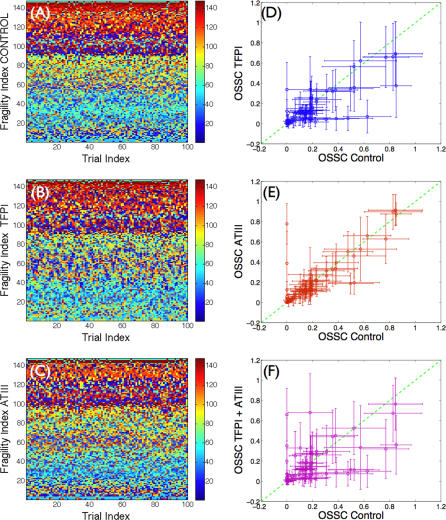
Sensitivity Analysis of the Coagulation Cascade OSSC values were calculated using randomly generated parameter sets constructed by perturbing the nominal parameter set by up to ±50% for each parameter (*N* = 100). (A–C) The *x*-axis denotes the trial index (index of the random parameter set), while the *y*-axis denotes the fragility index. The fragility index is calculated by determining the parameter index of the rank-ordered the OSSC values (the parameter index corresponding to the most fragile parameter has fragility index of 1; the next fragile is 2, while the most robust parameter has a fragility index of 148). The fragility index shows the robustness of a parameter; the smaller the fragility index, the more fragile the parameter. The parameter types are color-coded (shown in the color bar) and organized by biological function: 1–16, subendothelium interactions; 17–40, plasma interactions; 41–62, platelet surface binding; 63–77, platelet activation; 78–107, reactions on platelet surface; and 108–148, inhibitory reactions. (D–F) The OSSC values from the TFPI, ATIII, and TFPI + ATIII cases versus the control.

Simulations of TF–FVIIa initiated coagulation in the presence and absence of anticoagulants were compared with 21 published datasets from two different in vitro coagulation models [31,40,41]. The model parameters used in the validation simulations (unless otherwise noted) were compiled from literature and are shown in Table 1. Initial conditions for the validation simulations are given in the Protocol S1. To gauge the robustness and fragility of each interaction in the cascade, overall state sensitivity coefficients (OSSCs) were calculated for each of the 148 model parameters over a family of random parameter sets (see Methods and Materials). Simulation results are shown in Figure 2, and error quantification is reported in Table 2. With the exception of PC and one TFPI case, the model explained the time-resolved thrombin generation profile following TF–FVIIa addition to quiescent synthetic plasma. Platelet activation was assumed to be instantaneous for the synthetic plasma simulations. To test the ability of the model to simultaneously describe platelet activation and thrombin formation, simulations of TF–FVIIa initiated coagulation were compared with the in vitro cell-based assay of Roberts and coworkers [40] (Figure 2F); both platelet activation and thrombin generation profiles were consistent with the cell-based assay after adjusting three parameters used in the synthetic plasma simulations. Analysis of the sensitivity results for the control (no inhibitors) revealed that thrombin formation is controlled by both initiation and amplification mechanisms; the 25 most fragile mechanisms for the control are reported in Table 3, and the rank-ordered fragility results for 100 random parameter sets for the control, TFPI, and ATIII cases are shown in Figure 3A–3C. Mechanisms involving fluid and surface phase fX/FXa and thrombin were found to be the most sensitive, while mechanisms involving fIX/FIXa and fVIII/FVIIIa were found to be robust. Binding interactions were found to be the most sensitive group of interactions. Analysis of the significant shifts in the overall state sensitivity coefficients (see Methods and Materials) revealed both additive and synergistic effects when compared with the control (Figure 3).

**Table 2:**
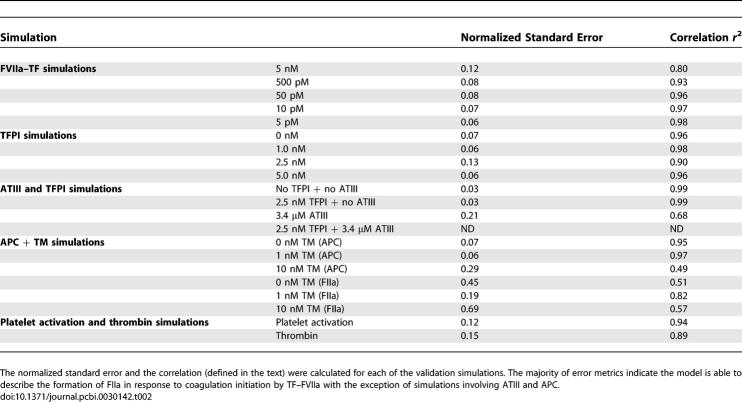
Quantification of Model Error

**Table 3:**
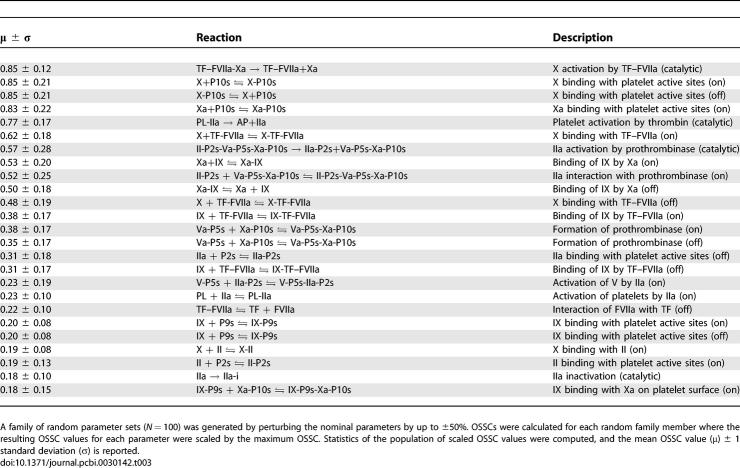
The 25 Most Fragile Coagulation Mechanisms in the Absence of Inhibitors

### Thrombin Activation in the Presence and Absence of Natural Anticoagulants

The predicted thrombin concentration profiles following the addition of TF–FVIIa to synthetic plasma were quantitatively consistent with in vitro observations (Figure 2E). The fraction of variation explained by the model (Table 2) was found to be inversely proportional to TF–FVIIa input strength. In the absence of inhibitor, thrombin generation was characterized by two regimes; at first, FXa generated thrombin in the bulk fluid, and then subsequently, the thrombin signal was amplified by activity of FVa–FXa surface complex (*prothrombinase*)*.* Decreasing the TF–FVIIa input prolonged the initiation phase as it slowed the rate of generation of FXa and thrombin in the bulk. However, TF–FVIIa input strength did not influence the maximal rate of thrombin formation because this is surface dominated; this was observed by comparing the slope or total net rate of thrombin generation across the TF–FVIIa cases.

TFPI and ATIII influenced both the initiation and amplification of the FIIa signal (Figure 2A and 2B). Increased TFPI concentration lead to a longer initiation delay and a decreased rate of FIIa amplification (Figure 2A). TFPI delayed initiation through interaction with free FXa and TF–FVIIa in an FXa-dependent manner; sequestering FXa reduced flux through the initial FXa-dependent route of FIIa generation. Thrombin amplification was negatively impacted by TFPI by reducing free FXa available for the formation of the FVa–FXa surface complex. On average, the model explained 95% of the TFPI dynamics; one exception was the 2.5 nM TFPI case (Figure 2A), where the correlation between model and experiment was 0.90. In contrast, ATIII reversibly binds FIXa, FXa, and TF–FVIIa, and irreversibly inactivates FIIa. ATIII produced a different thrombin generation profile when compared with TFPI (Figure 2B). ATIII influenced FIIa initiation by sequestering FXa (a mechanism similar to TFPI) and inactivated FIXa, thereby decreasing the rate of formation of the FVIIIa–FIXa surface complex. However, unlike TFPI, ATIII directly inactivated FIIa, leading to the decreasing FIIa concentration observed experimentally. While the model captured the qualitative features of ATIII (3.4 μM) activity, the correlation between the model and experiment was 0.68, indicating that slightly more than half of the FIIa dynamics were correctly described. The combination of TFPI (2.5 nM) and ATIII (3.4 μM), consistent with the experiment, completely quenched FIIa formation following TF–FVIIa addition.

Simulations of APC generation and inhibition of thrombin formation in the presence and absence of TM were qualitatively consistent with in vitro data (Figure 2C and 2D). APC was generated from PC via TM-dependent and -independent routes; the TM-dependent route catalyzes the conversion of PC to APC at a rate 400-fold greater than the thrombin route alone [41]. Three different TM concentrations were simulated, and the time course of APC and FIIa were compared with published data [41]. The correlation between model and experimental data for APC was approximately 96% for TM values ≤ 1 nM. However, the average correlation between the predicted and measured FIIa profile for TM ≤ 1 nM was 67%. Simulations of the TM 10 nM case failed to quantitatively capture both APC and FIIa formation; the correlation between model and experiment for APC in the presence of 10 nM TM was 49%, while 57% of the FIIa dynamics were correctly described.

In vivo, amplification of the thrombin signal requires the surface of activated platelets. Preliminary simulations of simultaneous platelet activation and thrombin formation (Figure 2F) were found to be consistent with the results of the cell-based coagulation assay of Roberts and coworkers [40]. The correlations between the measured and the simulated fraction of activated platelets and thrombin were 0.94 and 0.89, respectively. In contrast with coagulation in synthetic plasma, lag periods were observed for platelet activation and thrombin generation. Following the initial lag, the activated platelet concentration increases rapidly to a plateau of ∼100% activation. The thrombin concentration reaches a maximum of ∼55 nM at 30 min and then decreases to ∼0 nM at 70 min because of ATIII activity. Three parameter values were changed in the cell-based assay simulations compared with synthetic plasma; the rate constant controlling the activation of subendothelial bound platelets by thrombin was changed from 9.0 × 10^−3^ s^−1^ to 4.5 s^−1^, the activation of prothrombin by *prothrombinase* was changed from 30 s^−1^ to 6 s^−1^, and the binding of ATIII to thrombin was changed from 1.5 × 10^−5^ nM^−1^s^−1^ to 4.8 × 10^−5^ nM^−1^s^−1^.

### The Fragility and Robustness of the Coagulation Architecture

Overall state sensitivity coefficients were calculated for treatment cases A–D shown in Table 4. The top 25 fragile mechanisms for the control (case A) are shown in Table 3; four of the top five fragile mechanisms involve the binding or activation of fX/FXa, with the fifth being platelet activation by FIIa. Other sensitive mechanisms include the formation of the FVa–FXa surface complex, the activation of thrombin by the FVa–FXa complex, and the activation of surface or fluid phase fIX and fV. Fragility is spread across initiation and amplification mechanisms; 14 of 25 fragile mechanisms were upstream of the FVa–FXa or FVIIIa–FIXa surface complexes, while the remaining 11 involved platelet activation, FVa–FXa activity, and FIIa inactivation. Binding interactions were found to be the majority of fragile mechanisms; 21of 25 of the top fragile points were binding interactions. Six paired binding interactions were found to be sensitive, indicating affinity was controlling in these cases; the exception was the TF–FVIIa-X and Va-P5s-Xa-P10s complexes, where on rate, catalytic turnover, and off rate were all found to be sensitive. Of the predicted robust mechanisms, the most nonintuitive were the formation and activity of the FVIIIa–FIXa surface complex responsible for FXa amplification.

**Table 4:**
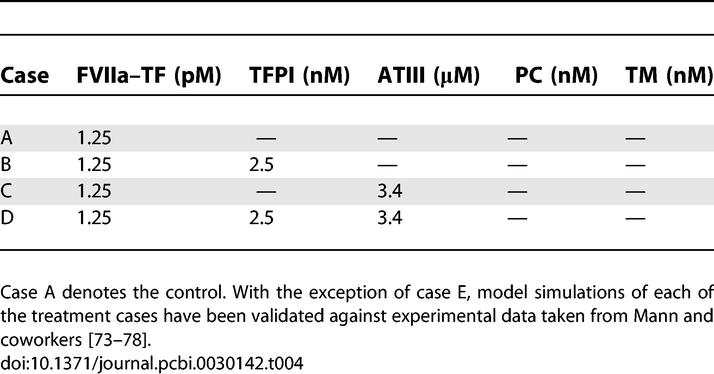
Treatment Cases Considered in the Sensitivity Analysis

Statistically significant changes in overall sensitivity coefficients relative to the control were used to gauge the importance of mechanisms in each treatment case. For example, if the sensitivity of a binding interaction increased relative to the control (became more fragile), then that interaction assumed increased importance in the treatment case. Conversely, a decrease in sensitivity relative to the control (mechanism became more robust) indicated a decrease of the overall impact of the mechanism. The anticoagulants TFPI and ATIII modulate thrombin formation by different mechanisms and have distinct regions of molecular influence. Although ATIII and TFPI share a common target (FXa), only two of 15 significant OSSC shifts were shared between the treatment cases (cases B and C) relative to the control (case A). TFPI was found to influence thrombin formation primarily through the FXa-specific interaction with TF–FVIIa; there were 13 mechanisms whose OSSC values changed significantly in response to TFPI (case A versus case B), nine of which become more robust, while two become more fragile (Figure 3B). TFPI reduced the fragility of the affinity of fIX and fX for TF–FVIIa, the sensitivity of TF–FVIIa-mediated formation of FXa, the affinity of FXa for fluid phase fIX, the stability of the TF–FVIIa complex, and the binding of fX and FXa with free-platelet binding sites. Conversely, TFPI increased the fragility of the off rate governing the disassociation of FXa from platelet binding sites and the interaction of itself with FXa. While ATIII had a more pronounced effect on FIIa generation than TFPI alone (Figure 2B), only four mechanisms were significantly affected by ATIII (case A versus case C). ATIII influenced thrombin formation through direct interaction with FXa and FIIa; ATIII reduced the sensitivity of the affinity of FXa for fIX in the fluid phase, while the on rate governing the binding of FXa and FIIa with ATIII was found to be of increased importance.

The predicted mechanism of action of anticoagulant combinations is not equivalent to the union of the individual treatment cases. The combination of TFPI + ATIII (case D) resulted in 14 statistically significant shifts relative to the control. Initiation mechanisms, e.g., the affinity of TF–FVIIa for fX and the affinity of FXa for fIX in the bulk fluid, were predicted to be less important. Conversely, the sensitivity of the off rate governing FXa interaction with the platelet surface was found to increase. Some amplification mechanisms became more robust, while others became fragile. The activation of platelets by FIIa via the protease-activated receptor family of surface receptors became less important, but the retention of FXa and the affinity of the surface FVa–FXa complex increased in importance. Last, trivial interactions resulting from the addition of TFPI and ATIII (the direct interactions of TFPI and TF–FVIIa in an FXa-dependent manner and ATIII with FXa) were predicted to become fragile relative to the control. Shifts in sensitivity coefficients were not additive across treatment cases; e.g., the compilation of significant shifts resulting from TFPI and ATIII addition was found not to be equivalent to the combination treatment. Of the 14 significant shifts observed in the TFPI + ATIII case (relative to the control), four mechanisms found to be sensitive in the individual cases were missing in the combination, while three novel shifts were observed. Interaction of fX/FXa with surface binding sites and the disassociation of the TF–FVIIa complex were found not to be significantly different than the control. The novel shifts in the TFPI + ATIII combination were all amplification mechanisms; the catalytic rate of IIa formation by FVa–FXa and the rate of platelet activation by IIa were found to be less important in the ATIII + TFPI combination, while interaction of surface-bound fV and FXa was found to increase in importance.

## Discussion

The predicted fragile mechanisms in the control are molecular targets in current anticoagulation preclinical development, clinical therapies, and clinical trials. Four of the top five fragile mechanisms involved fX/FXa or the activation of platelets by FIIa. Anti-fX/FXa and direct thrombin inhibitors (DTIs) are two important classes of anticoagulants (see Table 5 for a sampling of current clinical trials involving anti-fX/FXa strategies and DTIs). Fondaparinux, a 1.7 kDa pentasaccharide which selectively binds ATIII, is approved for the prevention of venous thromboembolism following hip fracture surgery, total hip replacement, total knee replacement, and major abdominal surgery in addition to the initial treatment of patients with deep venous thrombosis and pulmonary embolism [42–47]. Fondaparinux increases the natural inhibitory effect of ATIII against FXa approximately 300-fold [48,49]; selective inhibition of FXa by fondaparinux interrupts thrombin generation and clot formation without inactivating thrombin itself [50,51]. Elalamy and coworkers showed in a whole-blood in vitro assay that fondaparinux prolonged the lag time of prothrombin activation for all concentrations explored, and for physiologically relevant concentrations (0.11–0.28 anti-FXa IU/ml), reduced the maximal rate of thrombin formation to approximately 47%–55% of its nominal value [52]. Herbert and colleagues explored fondaparinux and the sulfated analog SANORG 32701 in in vivo mouse, rat, and rabbit coagulation models [53,54]; SANORG 32701 has a high affinity for ATIII (*K_d_* = 3.7 ± 0.7 nM) and shows more potent anti-FXa activity (1,100 ± 31 versus 850 ± 21 U/mg for fondaparinux). DTIs have also been explored as anticoagulants [55]. Thrombin activity is mediated by three protein domains: an active site catalyzing protease activity and two exosites controlling substrate binding [56]. Our sensitivity analysis predicts thrombin activation of platelets is a key mechanism; Sarich et al. have explored the DTI ximelagatran in healthy male volunteers [57]. Thrombin generation, platelet activation, and the thrombin–antithrombin complex were monitored in shed blood collected from skin incisions in 120 healthy male volunteers following oral administration of ximelagatran. Oral ximelagatran showed a rapid and statistically significant decrease in all endpoints relative to control. When taken together, the fondaparinux, SANORG 32701, and ximelagatran results present a clinical basis in both in vitro and in vivo coagulation studies for the prediction that fX/FXa and IIa are fragile components of the coagulation architecture.

**Table 5:**
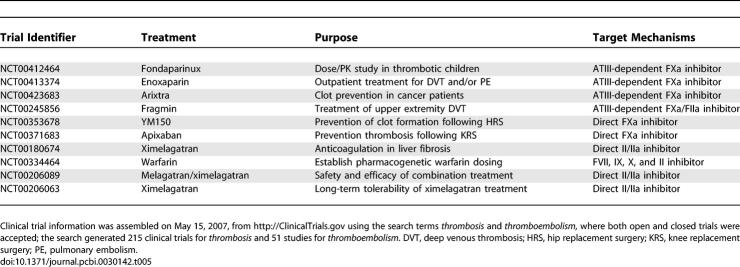
Ten Example Clinical Trials for FXa and Direct Thrombin Inhibitors

Mechanisms involving fIX/FIXa, consistent with multiple lines of experimental evidence, were predicted to be moderately robust. Feuerstein et al. explored inhibition of fIX/FIXa using a murine anti-fIX/FIXa antibody (BC2) in a male Sprague-Dawley rat model [58]. The fIX/FIXa activity and the activated partial thromboplastin time (aPTT) endpoints were monitored ex vivo following intravenous infusion of the BC2 antibody. Feuerstein et al. found that fIX/FIXa activity could be reduced by as much as 2.5-fold before any significant change in the aPTT was observed; only after >90% of the fIX/FIXa activity was eliminated was there a 3.5- to 4-fold increase in aPTT. Benedict et al. explored the contribution of fIX/FIXa to intravascular thrombosis in a canine coronary thrombosis model [59]. Animals received an intravenous bolus of saline (vehicle), bovine glutamyl-glycyl-arginyl-FIXa (FIXai; a competitive inhibitor which prevents the assembly of the FVIIIa–FIXa complex [60–62]), bovine fIX, or heparin. Animals that received saline or bovine fIX developed a coronary occlusion due to a fibrin/platelet thrombus in approximately 1 h; conversely, FIXai decreased coronary thrombus occlusion in a dose-dependent manner. However, FIXai administration was not accompanied by increased bleeding at abdominal and chest-wall incision sites, leading Benedict et al. to conclude, consistent with the earlier work of Weiss and Lages [63], that direct TF-FVIIa–mediated activation of fX may be the primary mechanism of fX activation in blood obtained from bleeding wounds. While our prediction that FVIIIa–FIXa-mediated fX activation is robust is consistent with Benedict et al., the robustness of fIX mechanisms should be further explored using in vivo animal models or cell-based assays to control for artifacts introduced by the synthetic plasma model.

Overall state sensitivity coefficients and shifts in sensitivity provide insight into the potential method of action of coagulation inhibitors, including synergistic effects, but are not predictors of clinical performance. The naive perspective that a specific inhibitor influences *only* its target and nothing else is not consistent with our sensitivity analysis. Consider the sensitivity results for TFPI; 13 different mechanisms were predicted to change significantly relative to the control. These shifts included not only the direct interactions of TFPI with FXa and TF–FVIIa, but also secondary effects like FXa interaction with fluid phase fIX. Moreover, the direction of shift, i.e., toward sensitivity or robustness, gives insight into the mechanism of action and the response of the network to the anticoagulant. In the case of TFPI, parameters associated with fluid phase FXa activity became more robust relative to the control, indicating these mechanisms were of decreased importance. Conversely, the off rate governing the disassociation of surface-bound FXa became more fragile relative to the control; keeping FXa bound became important as only fluid phase FXa binds TFPI in our model. However, while shifts in sensitivity coefficients might be a useful predictor of the network response, they are not predictors of clinical performance. No information about practical issues in patients, e.g., bioavailability, therapeutic window, unexpected toxicities, etc., was gained from sensitivity analysis of the network in isolation. Perhaps embedding the network into a pharmacokinetic or physiologically based pharmacokinetic (PBPK) model and then exploring the sensitivity profile of the augmented system could give insight into factors such as bioavailability and therapeutic window.

Despite parametric and structural uncertainty, the model captured the bulk of the thrombin generation dynamics resulting from TF–FVIIa-initiated coagulation in the presence and absence of natural anticoagulants. However, several challenges remain before the model is relevant to in vivo phenomena. First, pro- and anti-platelet activation mechanisms operating on the endothelium should be refined and/or included in the model. Predicted thrombin and APC concentrations in the presence of TM were not consistent with in vitro synthetic plasma measurements for TM concentrations >10 nM. The discrepancy between IIa and APC values remained despite changes in parameters indicating a potential structural issue with the model. Also not considered was the active role played by the endothelium; endothelial cells secrete anticoagulants (e.g., nitric oxide [NO] and prostacyclin) and express surface proteins (e.g., CD39). CD39 inhibits platelet activation by converting adenosine diphosphate, a potent inducer of platelet activation, into adenosine monophosphate [64]; however, CD39 may play a dual role, as Eniyoji et al. showed ΔCD39 mice had prolonged bleeding times and decreased platelet activation [65]. Second, the predicted fraction of activated platelets and the concentration of key complexes on the platelet surface need to be quantified under a variety of conditions. Initial simulations of simultaneous thrombin formation and platelet activation were consistent with the cell-based assay of Roberts and coworkers [40] (after changing three parameter values). However, these simulations and the cell-based assay were conducted assuming no flow and no regulatory input from the endothelium. Moreover, given the role that activated platelets play in the amplification of the thrombin signal, the predicted concentration of key complexes on the platelet surface, e.g., the FVIIIa–FIXa and FVa–FXa complexes, need to be validated. Third, the cell biology of coagulation and clot formation must be embedded in a description of physics occurring in clot formation. Several researchers have explored the role that blood flow plays upon the formation of clots. Antaki and coworkers developed a 2-D model of platelet deposition and activation in flowing blood [66]. The Antaki model was able to describe the axial platelet deposition on collagen under parallel-plate Poiseuille flow for a range of wall shear rates [67]. Diamond and coworkers have produced a rich body of work exploring the reaction complexity of human blood, cell aggregation, and adhesion under flow as well as the formation of key complexes on the surface of activated platelets under flow conditions, and established high-throughput techniques for real-time monitoring of coagulation dynamics. They have done stochastic modeling of the initiation of coagulation [68–72]. These literature sources (and others) will form the basis of our future development.

## Materials and Methods

### Formulation of the model equations.

The reactions considered in the coagulation model were compiled from literature and are given in Table 1. Mass balance equations were written around each protein or protein complex yielding the set of differential equations (vector form):

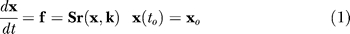
The symbol **S** denotes the stoichiometric matrix (92 × 148), while **x** denotes the concentration vector of proteins or protein complexes (92 × 1) and **r(x,k)** denotes the vector of reaction rates (148 × 1). Each row in **S** describes a particular protein or protein complex, while each column describes the stoichiometry associated with a specific interaction in the network. Thus, the (*i*,*j*) element of **S**, denoted by *σ_ij_*, describes how protein *i* is connected to rate process *j*. If *σ_ij_* < 0, then protein *i* is consumed in *r_j_*; conversely, if *σ_ij_* > 0, protein *i* is produced by *r_j_*, and if *σ_ij_* = 0, there is no connection between protein *i* and rate *j*. We have assumed mass action kinetics for each interaction in Table 1; under the mass action assumption, the rate expression for the general reaction *q*:

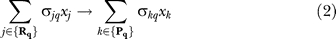
is given by:

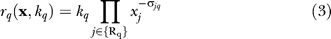
where {R*_q_*} denotes the set of reactants for reaction *q*, {P*_q_*} denotes the product set for reaction *q*, *k_q_* denotes the rate constant governing the *q*th reaction, and *σ_jq_*, *σ_kq_* denote stoichiometric coefficients (elements of the matrix **S**). We have treated every rate as nonnegative; all reversible reactions in Table 1 were split into two irreversible reaction steps. Thus, every element of the reaction rate vector **r(x,k)** takes the form shown in Equation 3.

The model equations were solved using the LSODE routine of the OCTAVE programming environment (http://www.octave.org; version 2.1.71) on an Apple Computer MacOSX (http://www.apple.com; v10.4.8) workstation. Model parameters and structure were compiled from literature; see Table 1 and [73–78]. Initial conditions were taken from each experiment and roughly correspond to in vivo physiological conditions (see Protocol S1). While the model presented here was developed from literature (including other models), it is, to the best of our knowledge, the only coagulation model to simultaneously describe all 21 datasets, including coupled platelet and thrombin activation and the activity of three different anticoagulants, using only minimal parameter variation (three-parameter change for a single case).

### Error analysis of the coagulation simulations.

The correlation between model simulations and experimental data and the scaled standard error were used to quantify the simulation uncertainty. The correlation between simulation and experimental observation was calculated using the relationship:

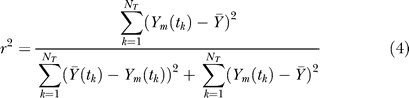
where *Y_m_*(*t_k_*) denotes the model value at time point *k*, 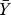 denotes the global average experimental value (average of experimental measurements over time), 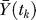 denotes the average experimental value at time point *k* (average of experimental trials at a single time point), and *N_T_* denotes the number of time points. The numerator of Equation 4 is the variation in the experimental data explained by the model, while the denominator is the total variation; thus, Equation 4 describes the fraction of the dynamics explained by the model across all time points. In addition to correlation, the scaled standard error was used to measure the agreement between the model and experiment:

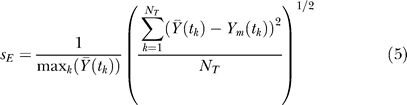
Both error metrics were taken from Spiegel [79].

### Computation of the OSSCs.

The sensitive or fragile elements of the coagulation architecture were determined by computing OSSCs [26]. Because each parameter corresponds directly to a particular molecular interaction in the cascade, OSSC values were used to gauge which elements of the architecture influence thrombin formation; large OSSC values for parameters relative to their peers indicates fragility or sensitivity, while small OSSC values indicates robustness. OSSC values were calculated by first calculating the first-order sensitivity coefficients:

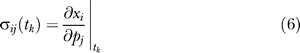
which are solutions of the differential equation:

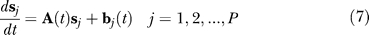
subject to the initial condition **s** *_j_*(*t*_0_) = **0**. In Equation 7, the quantity *j* denotes the parameter index, *P* denotes the number of parameters, **A** denotes the Jacobian matrix, and **b** *_j_* denotes the *j*th column of the matrix of first-derivatives of the mass balances with respect to the parameter values. The Jacobian matrix (**A**) and the matrix of first-derivatives of the mass balances with respect to the parameter values (**B**) are given by:

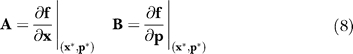
where (x^*^,p^*^) denotes a point along the *nominal* or *unperturbed* system solution. We solved Equation 7 for each parameter using the ODE15s routine of Matlab R2006a (The Mathworks, http://www.mathworks.com). The matrices **A** and **B** were estimated at each time step using a generalized gradient algorithm [80]. The OSSC value for parameter *j* defined as

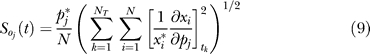
was computed using the scaled first-order sensitivities. The quantity *N_T_* denotes the number of time points used in the simulation, while *N* denotes the number of proteins/protein complexes in the model. To account for parametric uncertainty, the OSSC values (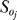 ) were calculated over a *family* of random parameter sets; we randomly perturbed each nominal parameter by up to ±≤50%, then solved the sensitivity balances for *each* family member. OSSC values were also computed over a family of random parameter sets generated by perturbing the nominal set by up to ±2 orders of magnitude. The large perturbation family produced similar results to those reported here (see Protocol S1).

### Statistical analysis of the shifts in OSSCs.

Two different statistical tests were performed to identify large statistically significant shifts in the OSSC values between treatment cases. A Welch *t*-test [81] was used to find all statistically significant shifts resulting from the different treatments, and then a secondary test on the *z*-score of each shift was performed to find only the most prominent significant shifts. The OSSC values calculated over the family of random parameter sets were assumed to follow normal distributions in each treatment case. The standard test to determine if the means of normal distributions are equal is the student *t*-test; however, the student *t*-test assumes the two distributions in question have equal variances. We cannot a priori guarantee this is true for the OSSC distributions in different treatment cases; thus, we have chosen the Welch *t*-test. The Welch *t*-test is very similar to the student *t*-test, with the exception that the two distributions being compared are not required to have equal variances. The statistical significance of shifts in OSSC values for each treatment case relative to the control were determined by performing a Welch *t*-test with the null hypothesis that the means of the OSSC values were equal at a 1% significance level. The list of significant OSSC values was further restricted to only those shifts with a magnitude larger than a specified *z*-score (0.1) away from the squared mean displacement over the significant OSSC values. We defined the displacement of an OSSC value relative to the control as:

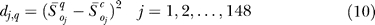
where 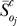 denotes the mean OSSC value over the family of random parameter sets for parameter *j* in the control, while 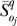 denotes the same quantity for treatment case *q*. A significant shift in OSSC value was *accepted* if:

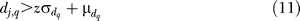
where *z* denotes a desired *z*-score, 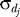 denotes the standard deviation of the total displacement over all significant OSSC values for the *q*th treatment case, and 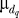 denotes the mean of the significant displacements for treatment case *q*.

## Supporting Information

Protocol S1 Impact of Perturbation Size on the Prediction of Fragile Mechanisms and the Initial Conditions for the Validation Simulations (156 KB PDF)

## Abbreviations

•-i: inactive form of species •
II: factor II or prothrombin
IIa: factor IIa or thrombin
V: factor V
VII: factor VII
VIIa: factor VIIa
VIIa–TF: factor Xa tissue factor complex
VIII: factor VIII
VIIIa–IXa: factor VIIIa IXa complex or tenase
IX: factor IX
IXa: factor IXa
X: factor X
Xa: factor Xa
AP: activated platelet
APC: activated protein C
aPTT: activated partial thromboplastin time
ATIII: antithrombin III
DTI: direct thrombin inhibitor
OSSC: overall state sensitivity coefficient
P★s: platelet surface binding sites for factor ★ or ★a, e.g., P2s represents platelet surface binding sites for factor II or IIa
P9s: IXa-specific binding sites on platelet surface
PC: protein C
Psub: subendothelium bind sites for resting and activated platelets
RP: resting platelet
TF: tissue factor
TFPI: TF pathway inhibitor
TM: thrombomodulin
Va: factor Va
