# Supplementary material for: Computationally Derived Points of Fragility of a Human Cascade Are Consistent with Current Therapeutic Strategies
Source: PLoS Comput Biol. 2007 Jul 20;3(7):e142. doi: 10.1371/journal.pcbi.0030142 (PMC1924874; doi:10.1371/journal.pcbi.0030142)
Supplement: Supplementary file 1 [file pcbi.0030142.sd001.pdf]

Protocol S1

Impact of perturbation size on the prediction of fragile mechanisms. The impact of perturbation size upon the classification of mechanisms as robust or fragile was explored by constructing a family of parameters generated by randomly perturbing each nominal parameter by up to ±2-orders of magnitude. Overall State Sensitivity Coefficients (OSSC) were calculated for each mechanism in the model over the family of random parameter sets ($N = 200$). Comparison of OSSC values obtained for the small (up to ±50%) versus large perturbation family (up to ±2-orders of magnitude) for the four treatment cases presented in the paper are shown in Fig. S1 (top). Several OSSC values are statistically different between the small and large perturbation families as determined by the Welch t-test, i.e., many OSSC values do not lie on the 45° line. However, the Spearman rank denoted by ρ and defined as:

$$\rho = 1 - \frac{6\sum_{i=1}^{P} d_i^2}{n\left(n^2-1\right)} \tag{1}$$

where d_i denotes the difference in the ordinal rank of parameter i between the small versus large perturbation cases and n denotes the number of pairs of values, shows an average of 96.6% agreement across treatment cases (see Fig. S1, bottom). Thus, while the numerical OSSC values are the not the same in the small versus large perturbation cases, the relative order of importance of the parameters is preserved. Therefore, the conclusions of this study would have been similar between small and large perturbations.

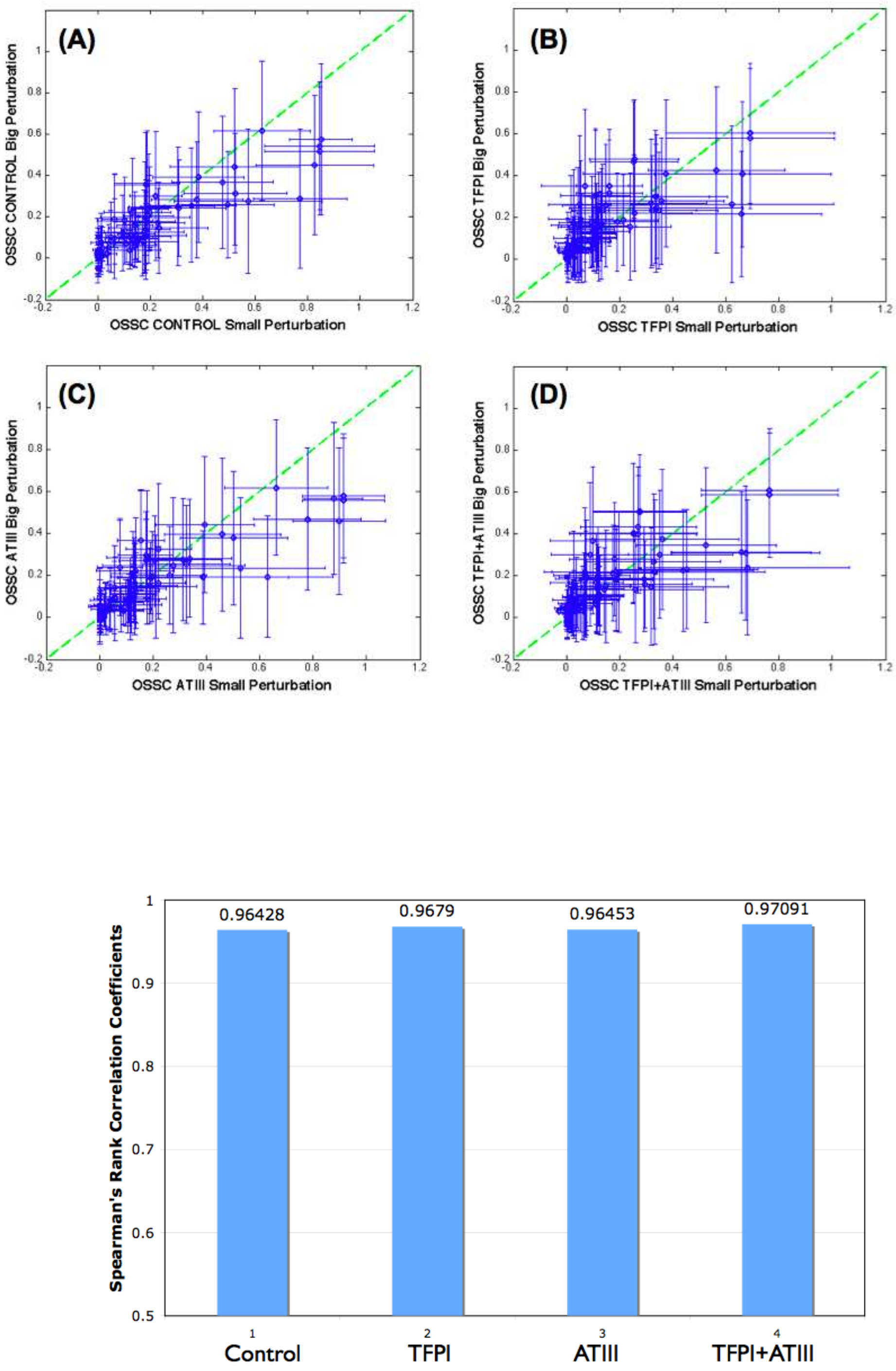

Figure S1: Sensitivity analysis results for large parameter perturbations. Top panel: Overall State Sensitivity Coefficients (OSSC) for the small versus large perturbation families for the different treatment cases considered. Bottom panel: Spearman rank correlation coefficients for the four treatment cases.

Table S1: Initial conditions for the validation simulations. The initial concentration of surface binding sites were determined so that the validation simulations were consistent with experimental observations, all other parameters and initial conditions held constant.

cases	A	B	C and D	E	F
fII	1700/2000 nM	1700 nM	1400 nM	1400 nM	1400 nM
fVII	–	–	–	–	10 nM
FVIIa	–	–	–	–	0.5 nM
TF	–	–	–	–	1 pM
TF-FVIIa	1.25 pM	1.25 pM	1.25 pM	5/10/50/500/5000 pM	–
fV	20 nM	20 nM	20 nM	20 nM	20 nM
fVIII	0.7 nM	0.7 nM	0.7 nM	0.7 nM	0.3 nM
fIX	90 nM	90 nM	90 nM	90 nM	70 nM
fX	170 nM	170 nM	170 nM	170 nM	135 nM
TFPI	0/1/2.5/5 nM	0/2.5 nM	2.5 nM	–	3 nM
ATIII	–	0/3400 nM	–	–	3000 nM
PC	–	–	65 nM	–	–
TM	–	–	0/1/10 nM	–	–
PL (fM)	150	150	150	150	150
PL binding sites on subendothelium (fM)	100	100	100	100	100
fII/FIIa binding sites (nmol/fmol-active platelets)	50	50	50	20	0.5
fV/FVa binding sites (nmol/fmol-active platelets)	50	50	50	20	50
fVIII/FVIIIa binding sites (nmol/fmol-active platelets)	5×10^{-10}	5×10^{-10}	5×10^{-10}	5×10^{-10}	5×10^{-6}
fIX/FIXa binding sites (nmol/fmol-active platelets)	50	50	50	20	5×10^{-8}
Specific fIX/FIXa binding sites (nmol/fmol-active platelets)	50	50	50	20	5×10^{-8}
fX/FXa binding sites (nmol/fmol-active platelets)	50	50	50	20	150
